# 2-Phenyl-5,7-bis­(prop-2-en-1-yl­oxy)-4*H*-chromen-4-one

**DOI:** 10.1107/S1600536808039743

**Published:** 2008-12-03

**Authors:** Angannan Nallasivam, Munirathinam Nethaji, Nagarajan Vembu, Venkatraman Ragunathan, Nagarajan Sulochana

**Affiliations:** a Department of Chemistry, National Institute of Technology, Tiruchirappalli 620 015, India; b Department of Inorganic and Physical Chemistry, Indian Institute of Science, Bangalore 560 012, India; c Department of Chemistry, Urumu Dhanalakshmi College, Tiruchirappalli 620 019, India; d Department of Chemistry, Kandasamy Kandar College, Velur 638 182, India

## Abstract

In the title compound, C_21_H_18_O_4_, tthe dihedral angle between the chromene ring system and the pendant phenyl ring is 6.1 (1)°. The crystal structure is stabilized by an intermolecular C—H⋯O and C—H⋯π inter­actions.

## Related literature

For the biological and pharmacological properties of benzopyrans and their derivatives, see Brooks (1998 ▶); Hatakeyama *et al.* (1988 ▶); Hyana & Saimoto (1987 ▶); Tang *et al.* (2007 ▶). For a detailed account of the importance of 4*H*-chromenes, see Liu *et al.* (2007 ▶); Wang, Fang *et al.* (2003 ▶); Wang, Zhang *et al.* (2003 ▶). For hydrogen-bonding inter­actions and motifs, see: Bernstein *et al.* (1995 ▶); Desiraju (1989 ▶); Desiraju & Steiner (1999 ▶); Etter (1990 ▶).
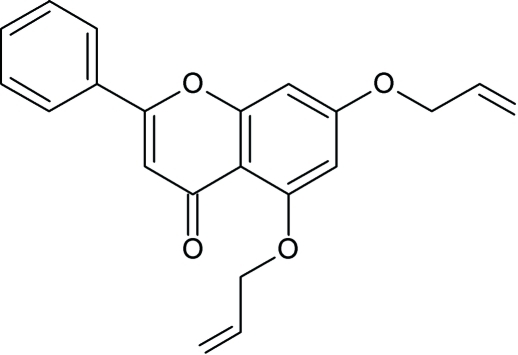


## Experimental

### 

#### Crystal data

C_21_H_18_O_4_
*M* *_r_* = 334.35
Orthorhombic, 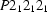

*a* = 6.299 (2) Å
*b* = 15.798 (6) Å
*c* = 17.429 (6) Å
*V* = 1734.3 (11) Å^3^
*Z* = 4
Mo *K*α radiation
μ = 0.09 mm^−1^
*T* = 293 (2) K
0.35 × 0.32 × 0.29 mm

#### Data collection

Bruker SMART APEX CCD diffractometer
Absorption correction: multi-scan (*SADABS*; Sheldrick, 1998 ▶) *T*_min_ = 0.969, *T*_max_ = 0.975
13979 measured reflections
2055 independent reflections
1793 reflections with *I* > 2σ(*I*)
*R*_int_ = 0.062

#### Refinement

*R*[*F* ^2^ > 2σ(*F* ^2^)] = 0.046
*wR*(*F* ^2^) = 0.098
*S* = 1.01
2055 reflections
226 parameters
H-atom parameters constrained
Δρ_max_ = 0.10 e Å^−3^
Δρ_min_ = −0.10 e Å^−3^

### 

Data collection: *SMART* (Bruker, 2007 ▶); cell refinement: *SMART*; data reduction: *SAINT* (Bruker, 2007 ▶); program(s) used to solve structure: *SHELXS97* (Sheldrick, 2008 ▶); program(s) used to refine structure: *SHELXL97* (Sheldrick, 2008 ▶); molecular graphics: *PLATON* (Spek, 2003 ▶); software used to prepare material for publication: *SHELXL97*.

## Supplementary Material

Crystal structure: contains datablocks I, global. DOI: 10.1107/S1600536808039743/fb2126sup1.cif

Structure factors: contains datablocks I. DOI: 10.1107/S1600536808039743/fb2126Isup2.hkl

Additional supplementary materials:  crystallographic information; 3D view; checkCIF report

## Figures and Tables

**Table 1:** Hydrogen-bond geometry (Å, °)

*D*—H⋯*A*	*D*—H	H⋯*A*	*D*⋯*A*	*D*—H⋯*A*
C17—H17*B*⋯O11^i^	0.97	2.51	3.229 (4)	131

Symmetry codes: (i) 
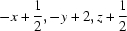
.

**Fig. 1.**
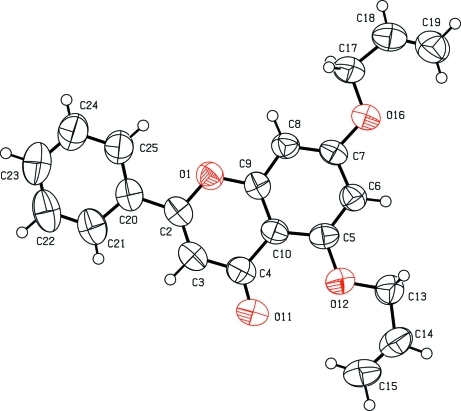
The title molecule showing the displacement ellipsoids depicted at the 50% probability level for all non-H atoms. The hydrogen atoms are drawn as spheres of arbitrary radius.

**Fig. 2.**
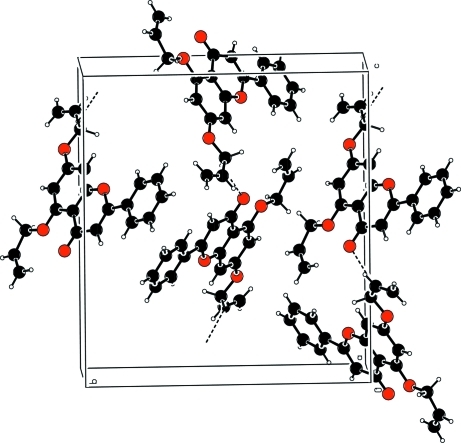
The molecular packing viewed down the c-axis. Dashed lines represent weak C–H···O interactions.

## supplementary crystallographic information

### Comment

Chromenes (benzopyrans) and their derivatives have numerous biological and pharmacological properties (Tang *et al.*, 2007) such as antisterility (Brooks, 1998) and anticancer activity (Hyana & Saimoto, 1987). In addition, polyfunctional chromene units are present in numerous natural products (Hatakeyama *et al.*, 1988). 4*H*-chromenes are important synthons for some natural products (Liu *et al.*, 2007). As a part of our structural investigations on 4*H*-chromene derivatives, the single-crystal X-ray diffraction study on the title compound was carried out.

The chromene ring is almost planar: The puckering amplitude of the chromene ring is 0.097 (3) Å. In the related chromene derivatives (Wang, Zhang *et al.*, 2003; Wang, Fang *et al.*, 2003), the chromene ring is also planar.  In the title structure, the interplanar angle between the chromene ring and the 2-phenyl ring is 6.1 (1)° thereby indicating their almost coplanar arrangement (Fig. 1). The propenyloxy substituents at both C5 and C7 are coplanar with the chromene ring with the respective interplanar angles 1.7 (2)° and 8.8 (2)°.

The crystal structure is stabilized by the interplay of C–H···O and C–H···π interactions (Fig. 2, Table 1; Desiraju & Steiner, 1999; Desiraju, 1989). Each of C15–H15A···O12, C19–H19A···O16 and C25–H25···O1 interactions are involved in the S(5) motifs (Bernstein *et al.*, 1995; Etter, 1990).

### Experimental

A suspension of chrysin (3.93 mmol, 1.00 g) and potassium carbonate (11.81 mmol, 1.64 g) in dimethyl formamide (10 ml) were added into a round bottom flask. The reaction mixture was heated to 383 K for 2–3 h. The reaction mixture was then cooled to 333 K and allyl bromide (15.74 mmol, 1.90 g) was slowly added to the reaction mixture with the help of a dropping funnel. The reaction mixture was maintained for 8–9 h at 333 K and monitored by high pressure liquid chromatography (HPLC). After completion of the reaction, the content was quenched with water and stirred for 30–45 min at 303 K. T he obtained crude solid was filtered and washed with plenty of water followed by methanol and dried under vacuum at 343 K. The compound was purified by column chromatography using ethyl acetate/n-hexane (1:1) as eluent. All highly pure column fractions were concentrated in a rota evaporator. The dried compound was dissolved in dichloromethane/hexane (1:1) mixture (10 ml). The clear solution was kept for a week and the resulting needle shaped crystals of average size 0.3 mm were washed with n-hexane. The crystals were dried over high vacuum at 343–348 K. Yield: 90%

### Refinement

In the absence of significant anomalous scattering effects, 1488 Friedel pairs have been merged. All the H-atoms were observed in the difference electron density map. However, they were situated into idealized positions with C–H = 0.93 and 0.97 Å for aryl and methylene H, respectively, and constrained to ride on their parent atoms with *U*_iso_(H) = 1.2*U*_eq_(C).

### Crystal data

**Table:**

C_21_H_18_O_4_	*D*_x_ = 1.281 Mg m^−^^3^
*M**_r_* = 334.35	Melting point = 434–437 K
Orthorhombic, *P*2_1_2_1_2_1_	Mo *K*α radiation, λ = 0.71073 Å
Hall symbol: P 2ac 2ab	Cell parameters from 576 reflections
*a* = 6.299 (2) Å	θ = 1.8–26.0°
*b* = 15.798 (6) Å	µ = 0.09 mm^−^^1^
*c* = 17.429 (6) Å	*T* = 293 K
*V* = 1734.3 (11) Å^3^	Needle, colourless
*Z* = 4	0.35 × 0.32 × 0.29 mm
*F*(000) = 704	

### Data collection

**Table:**

Bruker SMART APEX CCD diffractometer	3543 independent reflections
Radiation source: fine-focus sealed tube	1793 reflections with *I* > 2σ(*I*)
graphite	*R*_int_ = 0.062
Detector resolution: 0.3 pixels mm^-1^	θ_max_ = 26.4°, θ_min_ = 1.7°
ω scans	*h* = −7→7
Absorption correction: multi-scan (*SADABS*; Sheldrick, 1998)	*k* = −19→19
*T*_min_ = 0.969, *T*_max_ = 0.975	*l* = −21→20
13979 measured reflections	

### Refinement

**Table:**

Refinement on *F*^2^	Primary atom site location: structure-invariant direct methods
Least-squares matrix: full	Secondary atom site location: difference Fourier map
*R*[*F*^2^ > 2σ(*F*^2^)] = 0.046	Hydrogen site location: difference Fourier map
*wR*(*F*^2^) = 0.098	H-atom parameters constrained
*S* = 1.01	*w* = 1/[σ^2^(*F*_o_^2^) + (0.0343*P*)^2^ + 0.1512*P*] where *P* = (*F*_o_^2^ + 2*F*_c_^2^)/3
2055 reflections	(Δ/σ)_max_ < 0.001
226 parameters	Δρ_max_ = 0.10 e Å^−^^3^
0 restraints	Δρ_min_ = −0.10 e Å^−^^3^
72 constraints	

### Special details

**Table:**

Geometry. All e.s.d.'s (except the e.s.d. in the dihedral angle between two l.s. planes) are estimated using the full covariance matrix. The cell e.s.d.'s are taken into account individually in the estimation of e.s.d.'s in distances, angles and torsion angles; correlations between e.s.d.'s in cell parameters are only used when they are defined by crystal symmetry. An approximate (isotropic) treatment of cell e.s.d.'s is used for estimating e.s.d.'s involving l.s. planes.
Refinement. Refinement of *F*^2^ against ALL reflections. The weighted *R*-factor *wR* and goodness of fit *S* are based on *F*^2^, conventional *R*-factors *R* are based on *F*, with *F* set to zero for negative *F*^2^. The threshold expression of *F*^2^ > σ(*F*^2^) is used only for calculating *R*-factors(gt) *etc*. and is not relevant to the choice of reflections for refinement. *R*-factors based on *F*^2^ are statistically about twice as large as those based on *F*, and *R*- factors based on ALL data will be even larger.

### Fractional atomic coordinates and isotropic or equivalent isotropic displacement parameters (Å^2^)

**Table:**

	*x*	*y*	*z*	*U*_iso_*/*U*_eq_	
O1	0.4396 (4)	1.06971 (12)	0.13245 (11)	0.0650 (6)	
C2	0.5860 (6)	1.0832 (2)	0.0765 (2)	0.0639 (8)	
C3	0.5782 (6)	1.0403 (2)	0.0102 (2)	0.0745 (9)	
H3	0.6761	1.0541	−0.0278	0.089*	
C4	0.4277 (6)	0.9746 (2)	−0.0056 (2)	0.0696 (9)	
C5	0.0891 (6)	0.90891 (19)	0.04852 (18)	0.0608 (8)	
C6	−0.0584 (6)	0.90372 (18)	0.10630 (18)	0.0622 (8)	
H6	−0.1728	0.8668	0.1020	0.075*	
C7	−0.0358 (6)	0.95419 (19)	0.17160 (18)	0.0590 (8)	
C8	0.1332 (5)	1.00863 (19)	0.17997 (18)	0.0610 (9)	
H8	0.1495	1.0414	0.2239	0.073*	
C9	0.2778 (5)	1.01254 (18)	0.12026 (18)	0.0557 (8)	
C10	0.2641 (5)	0.96435 (18)	0.05336 (17)	0.0559 (8)	
O11	0.4392 (5)	0.93093 (17)	−0.06419 (14)	0.1049 (9)	
O12	0.0781 (4)	0.86278 (13)	−0.01724 (12)	0.0748 (6)	
C13	−0.0923 (6)	0.8053 (2)	−0.02818 (18)	0.0778 (10)	
H13A	−0.2267	0.8353	−0.0279	0.093*	
H13B	−0.0942	0.7635	0.0125	0.093*	
C14	−0.0582 (8)	0.7632 (2)	−0.1044 (2)	0.0860 (11)	
H14	−0.1579	0.7232	−0.1196	0.103*	
C15	0.0989 (7)	0.7778 (2)	−0.15084 (19)	0.0906 (12)	
H15A	0.2021	0.8173	−0.1378	0.109*	
H15B	0.1080	0.7487	−0.1971	0.109*	
O16	−0.1951 (4)	0.94477 (13)	0.22389 (13)	0.0729 (7)	
C17	−0.1985 (6)	1.0013 (2)	0.28825 (18)	0.0773 (11)	
H17A	−0.1929	1.0594	0.2704	0.093*	
H17B	−0.0753	0.9912	0.3204	0.093*	
C18	−0.3953 (7)	0.9877 (2)	0.3335 (2)	0.0815 (11)	
H18	−0.4206	1.0255	0.3733	0.098*	
C19	−0.5312 (6)	0.9305 (3)	0.3237 (2)	0.1011 (13)	
H19A	−0.5136	0.8910	0.2846	0.121*	
H19B	−0.6496	0.9277	0.3555	0.121*	
C20	0.7460 (6)	1.1464 (2)	0.0988 (2)	0.0670 (9)	
C21	0.9252 (7)	1.1595 (2)	0.0537 (2)	0.0818 (11)	
H21	0.9425	1.1292	0.0084	0.098*	
C22	1.0767 (7)	1.2170 (3)	0.0757 (3)	0.0980 (13)	
H22	1.1961	1.2253	0.0452	0.118*	
C23	1.0540 (8)	1.2625 (3)	0.1423 (3)	0.1075 (15)	
H23	1.1572	1.3015	0.1568	0.129*	
C24	0.8787 (8)	1.2503 (3)	0.1873 (3)	0.1096 (14)	
H24	0.8634	1.2806	0.2327	0.131*	
C25	0.7248 (6)	1.1932 (2)	0.1657 (2)	0.0887 (11)	
H25	0.6052	1.1859	0.1962	0.106*	

### Atomic displacement parameters (Å^2^)

**Table:**

	*U*^11^	*U*^22^	*U*^33^	*U*^12^	*U*^13^	*U*^23^
O1	0.0626 (14)	0.0716 (14)	0.0608 (14)	−0.0033 (13)	0.0010 (14)	0.0035 (11)
C2	0.059 (2)	0.066 (2)	0.067 (2)	0.008 (2)	0.003 (2)	0.0157 (18)
C3	0.071 (2)	0.084 (2)	0.069 (2)	−0.004 (2)	0.016 (2)	0.007 (2)
C4	0.067 (2)	0.079 (2)	0.063 (2)	0.003 (2)	0.006 (2)	0.0007 (19)
C5	0.072 (2)	0.0608 (19)	0.050 (2)	0.007 (2)	−0.003 (2)	0.0017 (16)
C6	0.066 (2)	0.0620 (18)	0.058 (2)	−0.0021 (18)	0.003 (2)	0.0036 (17)
C7	0.062 (2)	0.0673 (19)	0.048 (2)	0.0043 (19)	0.0072 (19)	0.0059 (17)
C8	0.067 (2)	0.0646 (19)	0.0509 (19)	0.0001 (18)	0.001 (2)	0.0015 (16)
C9	0.054 (2)	0.0572 (17)	0.056 (2)	0.0028 (18)	−0.0023 (19)	0.0097 (16)
C10	0.059 (2)	0.0577 (17)	0.051 (2)	0.0056 (19)	0.0027 (18)	0.0042 (15)
O11	0.107 (2)	0.128 (2)	0.0792 (18)	−0.029 (2)	0.0327 (17)	−0.0308 (16)
O12	0.0831 (16)	0.0798 (14)	0.0614 (15)	−0.0099 (15)	0.0049 (14)	−0.0105 (13)
C13	0.086 (3)	0.076 (2)	0.071 (2)	−0.013 (2)	0.002 (2)	−0.0011 (19)
C14	0.110 (3)	0.078 (2)	0.070 (3)	−0.008 (3)	−0.013 (3)	−0.010 (2)
C15	0.118 (3)	0.094 (3)	0.060 (2)	0.011 (3)	−0.004 (3)	−0.007 (2)
O16	0.0753 (17)	0.0844 (14)	0.0591 (14)	−0.0090 (13)	0.0117 (13)	−0.0065 (12)
C17	0.088 (3)	0.088 (2)	0.057 (2)	−0.004 (2)	0.010 (2)	−0.0027 (19)
C18	0.092 (3)	0.092 (3)	0.060 (2)	0.012 (3)	0.000 (3)	0.003 (2)
C19	0.082 (3)	0.125 (3)	0.096 (3)	0.003 (3)	0.010 (3)	−0.010 (3)
C20	0.061 (2)	0.0622 (19)	0.078 (3)	0.002 (2)	−0.004 (2)	0.0161 (19)
C21	0.070 (3)	0.084 (2)	0.092 (3)	0.000 (2)	−0.002 (3)	0.028 (2)
C22	0.072 (3)	0.095 (3)	0.127 (4)	−0.012 (3)	−0.002 (3)	0.044 (3)
C23	0.092 (3)	0.083 (3)	0.147 (5)	−0.021 (3)	−0.015 (4)	0.012 (3)
C24	0.096 (3)	0.100 (3)	0.132 (4)	−0.022 (3)	0.000 (3)	−0.019 (3)
C25	0.077 (3)	0.082 (2)	0.107 (3)	−0.010 (2)	0.005 (3)	−0.010 (2)

### Geometric parameters (Å, °)

**Table:**

O1—C2	1.360 (4)	C14—H14	0.9300
O1—C9	1.378 (3)	C15—H15A	0.9300
C2—C3	1.340 (4)	C15—H15B	0.9300
C2—C20	1.471 (5)	O16—C17	1.434 (3)
C3—C4	1.433 (5)	C17—C18	1.485 (5)
C3—H3	0.9300	C17—H17A	0.9700
C4—O11	1.233 (3)	C17—H17B	0.9700
C4—C10	1.465 (4)	C18—C19	1.256 (4)
C5—O12	1.360 (3)	C18—H18	0.9300
C5—C6	1.373 (4)	C19—H19A	0.9300
C5—C10	1.410 (4)	C19—H19B	0.9300
C6—C7	1.397 (4)	C20—C25	1.387 (4)
C6—H6	0.9300	C20—C21	1.391 (5)
C7—O16	1.364 (4)	C21—C22	1.373 (5)
C7—C8	1.376 (4)	C21—H21	0.9300
C8—C9	1.384 (4)	C22—C23	1.373 (5)
C8—H8	0.9300	C22—H22	0.9300
C9—C10	1.395 (4)	C23—C24	1.368 (6)
O12—C13	1.419 (4)	C23—H23	0.9300
C13—C14	1.501 (4)	C24—C25	1.377 (5)
C13—H13A	0.9700	C24—H24	0.9300
C13—H13B	0.9700	C25—H25	0.9300
C14—C15	1.299 (5)		
			
C2—O1—C9	119.6 (3)	C15—C14—H14	117.2
C3—C2—O1	121.0 (3)	C13—C14—H14	117.2
C3—C2—C20	126.6 (4)	C14—C15—H15A	120.0
O1—C2—C20	112.4 (3)	C14—C15—H15B	120.0
C2—C3—C4	123.8 (3)	H15A—C15—H15B	120.0
C2—C3—H3	118.1	C7—O16—C17	117.8 (3)
C4—C3—H3	118.1	O16—C17—C18	109.7 (3)
O11—C4—C3	121.7 (3)	O16—C17—H17A	109.7
O11—C4—C10	124.1 (3)	C18—C17—H17A	109.7
C3—C4—C10	114.2 (3)	O16—C17—H17B	109.7
O12—C5—C6	123.5 (3)	C18—C17—H17B	109.7
O12—C5—C10	115.0 (3)	H17A—C17—H17B	108.2
C6—C5—C10	121.5 (3)	C19—C18—C17	126.9 (4)
C5—C6—C7	119.6 (3)	C19—C18—H18	116.5
C5—C6—H6	120.2	C17—C18—H18	116.5
C7—C6—H6	120.2	C18—C19—H19A	120.0
O16—C7—C8	124.5 (3)	C18—C19—H19B	120.0
O16—C7—C6	114.0 (3)	H19A—C19—H19B	120.0
C8—C7—C6	121.5 (3)	C25—C20—C21	118.3 (4)
C7—C8—C9	117.2 (3)	C25—C20—C2	121.2 (3)
C7—C8—H8	121.4	C21—C20—C2	120.5 (3)
C9—C8—H8	121.4	C22—C21—C20	120.3 (4)
O1—C9—C8	113.6 (3)	C22—C21—H21	119.8
O1—C9—C10	122.1 (3)	C20—C21—H21	119.8
C8—C9—C10	124.3 (3)	C21—C22—C23	120.7 (4)
C9—C10—C5	115.9 (3)	C21—C22—H22	119.7
C9—C10—C4	118.9 (3)	C23—C22—H22	119.7
C5—C10—C4	125.2 (3)	C24—C23—C22	119.7 (4)
C5—O12—C13	119.6 (3)	C24—C23—H23	120.2
O12—C13—C14	107.1 (3)	C22—C23—H23	120.2
O12—C13—H13A	110.3	C23—C24—C25	120.2 (4)
C14—C13—H13A	110.3	C23—C24—H24	119.9
O12—C13—H13B	110.3	C25—C24—H24	119.9
C14—C13—H13B	110.3	C24—C25—C20	120.8 (4)
H13A—C13—H13B	108.5	C24—C25—H25	119.6
C15—C14—C13	125.6 (4)	C20—C25—H25	119.6
			
C9—O1—C2—C3	−1.5 (4)	O11—C4—C10—C9	174.5 (3)
C9—O1—C2—C20	179.6 (2)	C3—C4—C10—C9	−5.1 (4)
O1—C2—C3—C4	−4.0 (5)	O11—C4—C10—C5	−5.6 (5)
C20—C2—C3—C4	174.8 (3)	C3—C4—C10—C5	174.8 (3)
C2—C3—C4—O11	−172.6 (3)	C6—C5—O12—C13	−0.4 (4)
C2—C3—C4—C10	7.1 (5)	C10—C5—O12—C13	−179.7 (3)
O12—C5—C6—C7	−179.3 (3)	C5—O12—C13—C14	−178.8 (3)
C10—C5—C6—C7	−0.1 (5)	O12—C13—C14—C15	−0.3 (5)
C5—C6—C7—O16	178.4 (3)	C8—C7—O16—C17	6.5 (4)
C5—C6—C7—C8	−0.8 (5)	C6—C7—O16—C17	−172.7 (3)
O16—C7—C8—C9	−177.9 (3)	C7—O16—C17—C18	173.6 (3)
C6—C7—C8—C9	1.2 (4)	O16—C17—C18—C19	6.9 (5)
C2—O1—C9—C8	−176.4 (2)	C3—C2—C20—C25	173.0 (3)
C2—O1—C9—C10	3.2 (4)	O1—C2—C20—C25	−8.1 (4)
C7—C8—C9—O1	178.8 (2)	C3—C2—C20—C21	−8.2 (5)
C7—C8—C9—C10	−0.9 (4)	O1—C2—C20—C21	170.7 (3)
O1—C9—C10—C5	−179.5 (2)	C25—C20—C21—C22	0.4 (5)
C8—C9—C10—C5	0.1 (4)	C2—C20—C21—C22	−178.4 (3)
O1—C9—C10—C4	0.3 (4)	C20—C21—C22—C23	−0.1 (5)
C8—C9—C10—C4	179.9 (3)	C21—C22—C23—C24	0.2 (6)
O12—C5—C10—C9	179.7 (3)	C22—C23—C24—C25	−0.6 (7)
C6—C5—C10—C9	0.4 (4)	C23—C24—C25—C20	1.0 (6)
O12—C5—C10—C4	−0.1 (4)	C21—C20—C25—C24	−0.9 (5)
C6—C5—C10—C4	−179.4 (3)	C2—C20—C25—C24	178.0 (3)

### Hydrogen-bond geometry (Å, °)

**Table:**

*D*—H···*A*	*D*—H	H···*A*	*D*···*A*	*D*—H···*A*
C15—H15A···O12	0.93	2.35	2.691 (4)	101
C19—H19A···O16	0.93	2.42	2.749 (5)	101
C25—H25···O1	0.93	2.39	2.714 (4)	101
C17—H17B···O11^i^	0.97	2.51	3.229 (4)	131
C14—H14···Cg1^ii^	0.93	3.22	4.081	154

Symmetry codes: (i) −*x*+1/2, −*y*+2, *z*+1/2; (ii) −*x*−1, *y*+3/2, −*z*+1/2.
